# Bioethics for a burning planet: why Planetary Health and One Health might not be the way to go

**DOI:** 10.1080/11287462.2025.2593723

**Published:** 2025-12-08

**Authors:** Katharina Wabnitz, Bridget Pratt, Cristian Timmermann, Verina Wild

**Affiliations:** a Institute for Ethics and History of Health in Society, Medical Faculty, University of Augsburg, Augsburg, Germany; b Queensland Bioethics Centre, Australian Catholic University, Banyo, Queensland, Australia

**Keywords:** Bioethics, Planetary Health, One Health, indigenous knowledge

## Abstract

Climate change, ecological degradation and global inequalities are symptoms of an eco-social polycrisis that threatens global health and health equity. This polycrisis is deeply rooted in Western value systems. These can be described as anthropocentric and individualistic and support the prevailing neoliberal economic model. Bioethics is now called to respond to the urgent health-related ethical challenges of the polycrisis and has recently begun to engage with Planetary Health and One Health in this regard. Both have mainly emerged in the Western scientific community and understand human health to be inextricably linked to the state of environmental and structural societal determinants. We argue that bioethics should indeed embrace holistic or integrated understandings of health but also carefully revisit the foundational Western value systems at the root of the polycrisis. If Planetary Health and One Health stay grounded in Western value systems, an extensive conceptual engagement might be problematic for bioethics. Instead of turning to Western concepts of health, bioethics should engage deeply with Indigenous and non-Western ways of knowing and critically reflect on its own role in inadvertently maintaining the status quo.

At the time of writing, Europe is struck by major heatwaves and wildfires. Temperatures are yet again record-breaking. Earlier this year, Australia’s east coast and the USA’s south saw cyclones and heavy rainfall causing severe flooding. New records for low temperatures were recorded in Chile. Extreme weather events occur with increased intensity and frequency due to climate change (Vautard et al., 2023). Climate change and the ongoing transgression of ecological boundaries are only one part of an eco-social polycrisis affecting health and health equity: Material and immaterial resources to secure life, health and well-being are immensely unequally distributed within and between nations (Chancel et al., 2022) and dissent on the need for systemic change towards just societies on a healthy planet seems to permeate much of societal and global discourse. The search for ethically adequate solutions commensurate with the scale of the polycrisis becomes ever more urgent, with bioethics playing a role in identifying and discussing health-related aspects (Macpherson, 2013; Anderson et al., 2025). We explore whether an engagement of bioethics with One Health and Planetary Health is promising in this regard.

## The broader structures within which bioethics operates

Global health institutions and structures within which human activities unfold are often underpinned by Western values and norms. In bioethics and global health ethics these Western values and norms have been characterized as *individualistic*—valuing the individual over the whole, and *anthropocentric*—giving human interests priority over nature, based on a worldview that understands nature as instrumental for human wellbeing. In relation to global health justice, Solomon Benatar argued that the polycrisis is “the outcome of the way in which the global political economy has been restructured [.] and that this is causally related to a value system” (Benatar, 2013) which he describes as “distorted”. The values that protect the rights and freedoms of individuals are commonly seen as worth defending, but they have grown into versions of themselves that contribute to perpetuating the polycrisis: Benatar speaks of “hyper-individualism” and “narrow conceptions and distorted application of human rights” (Benatar, 2013). Relatedly, he and colleagues stated that in the neoliberal model of development a notion of freedom as “minimally restricted freedom of the individual and of enterprise” (Benatar et al., 2018) and of progress as self-evident prevail. In its logic of profit, the future is systematically discounted, and private goods have precedence over common goods. The negative impacts on health, wellbeing, livelihoods, and nature are evident. As Hensher et al. argued, economic growth can now be seen as uneconomic as it generates failure demand (“a cycle of paying to fix what we continue to break” (Hardt, 2022)) for healthcare (Hensher et al., 2020).

## Planetary Health and One Health as a way forward for bioethics?

Bioethics and environmental ethics evolved into two “distinct and distant fields” (Lee, 2017). Recently, however, there have been efforts to bridge the gap. Bioethics is currently finding its role in relation to environmental issues, including calling for broader structural change (Pratt, 2022; Wardrope, 2020; Wild, 2026). In these efforts, there have been calls to revisit the roots of bioethics (Anderson et al., 2025; Lee, 2017; ten Have, 2019), including its original conceptualization by Van Rensselaer Potter in the 1970’s. Potter highlights that “human ethics cannot be separated from a realistic understanding of ecology in the broadest sense. Ethical values cannot be separated from biological facts” (Potter, 1970). Bioethics has also begun to engage with Planetary Health and One Health. Both propose holistic concepts of health and recognize the interconnectedness of health and well-being with non-human animals, plants, ecosystems and natural systems such as the global climate. Planetary Health has been dubbed “a new science for exceptional action” by some of its early proponents (Horton and Lo, 2015). But do these concepts advance bioethics in an original way? Do they provide the starting point for a normative theory of change that could contribute to transforming the distorted value systems as described by Benatar et al. and resulting societal structures? Planetary Health and One Health both reflect a broad, eco-aware understanding of health. Some scholars have enriched these concepts by drawing from Indigenous knowledges (Hoogeveen et al., 2023; Redvers et al., 2022). Indigenous knowledges and value systems are commonly rooted in non-anthropocentric worldviews. But Planetary Health and One Health have emerged mainly from within the Western scientific community and adherents mostly seem to retain an anthropocentric stance (Cañada et al., 2022; Lerner and Berg, 2017; Bingham et al., 2025). Planetary Health has even been critiqued for contributing to (neo)colonial domination (Baquero et al., 2021). What is more, academic disciplines or movements that establish themselves within the prevailing value system by introducing seemingly new normative concepts may inadvertently reinforce the status quo. Even if their stated goal is to overturn or at least minimise harm within the prevailing system, they may ultimately, “perform[s] an implicit ideological function of legitimising and reproducing the existing power structure” (Kim, 2021) as Hani Kim stated in relation to Global Health. One Health and Planetary Health might thus be more recent examples of the same phenomenon. This could become problematic for bioethics if it is set to scrutinize Western values—a need that Alistair Campbell already emphasized in 1998 in his presidential address to the 4^th^ World Congress of Bioethics. He called upon the audience to critically reflect on bioethics’ tacit contribution to “an unimpeded advance of business” and to scrutinize (Western) norms which endorse “the idea of constant economic progress as an end for humanity” (Campbell, 1999).

## Bioethics needs to look outside Western knowledge and value systems

We conclude that if bioethics wants to live up to the challenges of this century, it will need to revisit foundational Western value systems and embrace a holistic understanding of health. For this, it is questionable what could be gained from extensive conceptual engagement with yet another Western concept of health such as Planetary Health or One Health as long as they remain rooted in Western value systems. Rather, as Whitehouse said, “the future of bioethics lies to a considerable degree in its past” (Whitehouse, 2003)—a call that others have supported (Lee, 2017; Richie, 2020). Furthermore, critically examining prevailing value systems and the neoliberal paradigm might free bioethics from the risk of involuntarily legitimising the status quo. This requires building awareness of the historical causes and effects of culturally induced ignorance originating from patriarchal and (neo-)colonial structures (Schiebinger, 2004). There remains a need to provide moral guardrails for current ethical challenges, e.g. in data-intensive clinical care, that arise within the present conditions. But to overcome the polycrisis, prevailing worldviews and value systems might have to be revisited much more explicitly and profoundly to ultimately yield societal structures that guide human activities back within planetary boundaries and ensure human health and wellbeing for all (Bingham et al., 2025; Whyte, 2020). Engaging with Indigenous and non-Western ways of knowing, morally valuing and conceiving of human wellbeing, with their longstanding understanding of a good life as being in harmony with the non-human environment, represent to us promising and necessary avenues for developing bioethics for the 21^st^ century.
